# Effect of Sub-Stoichiometric Fe(III) Amounts on LCFA Degradation by Methanogenic Communities

**DOI:** 10.3390/microorganisms8091375

**Published:** 2020-09-07

**Authors:** Ana J. Cavaleiro, Ana P. Guedes, Sérgio A. Silva, Ana L. Arantes, João C. Sequeira, Andreia F. Salvador, Diana Z. Sousa, Alfons J. M. Stams, M. Madalena Alves

**Affiliations:** 1 Centre of Biological Engineering, University of Minho, Campus de Gualtar, 4710-057 Braga, Portugal; guedesana@esa.ipvc.pt (A.P.G.); sergiosilva@ceb.uminho.pt (S.A.S.); analuisa.pereira@ceb.uminho.pt (A.L.A.); jsequeira@ceb.uminho.pt (J.C.S.); asalvador@ceb.uminho.pt (A.F.S.); diana.sousa@wur.nl (D.Z.S.); fons.stams@wur.nl (A.J.M.S.); madalena.alves@deb.uminho.pt (M.M.A.); 2 Laboratory of Microbiology, Wageningen University & Research, 6708 WE Wageningen, The Netherlands

**Keywords:** long chain fatty acids, oleate, Fe(III), methanogenesis, *Syntrophomonas*, *Geobacter*

## Abstract

Long-chain fatty acids (LCFA) are common contaminants in municipal and industrial wastewater that can be converted anaerobically to methane. A low hydrogen partial pressure is required for LCFA degradation by anaerobic bacteria, requiring the establishment of syntrophic relationships with hydrogenotrophic methanogens. However, high LCFA loads can inhibit methanogens, hindering biodegradation. Because it has been suggested that anaerobic degradation of these compounds may be enhanced by the presence of alternative electron acceptors, such as iron, we investigated the effect of sub-stoichiometric amounts of Fe(III) on oleate (C18:1 LCFA) degradation by suspended and granular methanogenic sludge. Fe(III) accelerated oleate biodegradation and hydrogenotrophic methanogenesis in the assays with suspended sludge, with H_2_-consuming methanogens coexisting with iron-reducing bacteria. On the other hand, acetoclastic methanogenesis was delayed by Fe(III). These effects were less evident with granular sludge, possibly due to its higher initial methanogenic activity relative to suspended sludge. Enrichments with close-to-stoichiometric amounts of Fe(III) resulted in a microbial community mainly composed of *Geobacter*, *Syntrophomonas*, and *Methanobacterium* genera, with relative abundances of 83–89%, 3–6%, and 0.2–10%, respectively. In these enrichments, oleate was biodegraded to acetate and coupled to iron-reduction and methane production, revealing novel microbial interactions between syntrophic LCFA-degrading bacteria, iron-reducing bacteria, and methanogens.

## 1. Introduction

Lipids and long-chain fatty acids (LCFA) are frequently present as contaminants in municipal and industrial wastewater [1]. Due to their hydrophobic character, these compounds are generally associated with sludge flotation and foaming in conventional aerobic wastewater treatment plants [2]. Furthermore, LCFA can inhibit microbial growth, especially when present in high concentrations [2]. Compared to aerobic treatment, anaerobic digestion (AD) of lipid/LCFA-rich wastewater has the advantage of generating biogas from these energy-rich compounds, and normally, higher LCFA loads can be applied [1]. In AD, lipids are hydrolyzed to glycerol and LCFA, which are further converted to acetate and hydrogen/formate by syntrophic bacteria. This reaction is thermodynamically feasible only when hydrogen/formate concentrations are kept low [3,4]. The activity of methanogenic archaea, which act as hydrogen/formate scavengers, is thus crucial for maintaining low concentrations of these intermediate compounds.

Biodegradation of lipids and LCFA has been extensively studied in methanogenic bioreactors [1,5], but limited information is available regarding similar processes in the presence of external electron acceptors, such as iron, nitrate, or sulfate. These can be important when methanogens are absent or inhibited, especially considering that methanogens are generally reported as highly sensitive to LCFA [6,7,8,9]. In particular, iron is frequently supplemented in wastewater treatment plants, namely for organic matter removal, phosphorous removal, or sulfide control [10,11,12], and thus, it may reach the anaerobic digestion step. In this work, addition of Fe(III) was studied as a strategy to promote oleate (C18:1 LCFA) biodegradation and counteract LCFA toxicity towards methanogens. With this strategy, process performance stability is aimed for, although it may constitute a trade-off at the expense of methane yield.

Degradation of LCFA by iron(III)-reducing bacteria (IRB) has been reported, by e.g., *Desulfuromonas palmitatis* [13] and *Geothrix fermentans* [14]. IRB can oxidize LCFA completely to CO_2_, or incompletely to acetate, by reducing Fe(III) to Fe(II) [15]. Most IRB are also able to utilize acetate, hydrogen, or formate as electron donors [16] and thus, their involvement in LCFA biodegradation as syntrophic partners of fatty-acid oxidizing acetogenic bacteria can be hypothesized. Fe(III) reduction results in lower hydrogen thresholds and is thermodynamically more favorable than hydrogenotrophic methanogenesis [17], which represents an advantage for syntrophic relationships.

The addition of sub-stoichiometric amounts of Fe(III) to methanogenic communities has been suggested to alleviate LCFA toxicity towards methanogens. Li et al. [18] reported that enriched cultures, developed in the presence of sub-stoichiometric amounts of ferric hydroxide, converted vegetable oil or a mixture of oleate and acetate faster to methane than the microbial communities that were developed without ferric hydroxide. Apparently, the enrichment with Fe(III) selected a microbial community less sensitive to LCFA inhibition, with IRB possibly replacing LCFA-sensitive acetoclastic methanogens and/or fatty acid oxidizing acetogens [18,19]. Additionally, Li et al. [20] reported accelerated methanogenesis from canola oil or oleate by anaerobic sludge from a municipal sludge digester in the presence of ferric hydroxide. In these studies, changes at the level of microbial composition were not assessed.

If methane production is intended, Fe(III) should be dosed to avoid out-competition of methanogens by IRB [21]. Moreover, amorphous Fe(OH)_3_ was shown to directly inhibit the activity of *Methanospirillum hungatei* and *Methanosaeta concilii* grown in pure culture [22], and some methanogens (e.g., *Methanosarcina barkeri*) also have the ability to reduce Fe(III), which may contribute to methanogenesis inhibition [22,23]. Bond and Lovley [24] reported that the inhibition of methane production in Fe(III) oxide-containing sediments may result, at least in part, from methanogens diverting electrons to Fe(III) reduction.

This work aims to study the effect of Fe(III) on methanogenic LCFA degradation and to identify the microbial key players involved. In an initial experiment, suspended and granular sludge were incubated with oleate at sub-stoichiometric ferric hydroxide concentrations to assess their potential to lower LCFA toxicity towards methanogens. Then, the microbial communities involved in LCFA biodegradation were characterized in enrichments containing close-to-stoichiometric Fe(III) concentrations.

## 2. Materials and Methods

### 2.1. Oleate Biodegradation with Sub-Stoichiometric Fe(III) Concentration by Anaerobic Sludge

**Figure 1:**
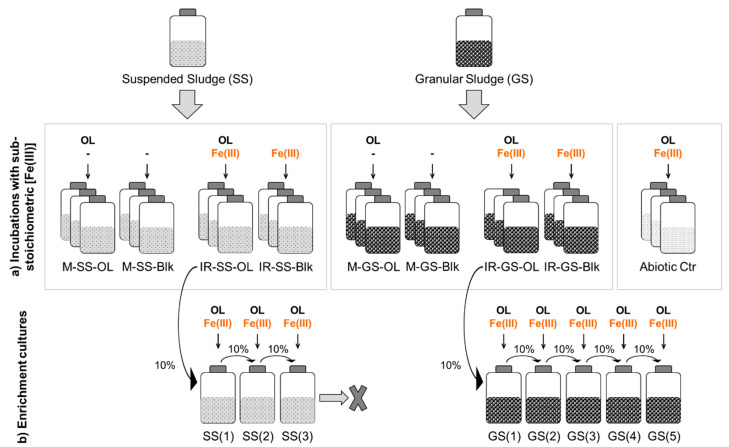
Scheme of the experimental procedure applied: oleate biodegradation with sub-stoichiometric Fe(III) concentration (**a**) and enrichment cultures (**b**). SS—suspended sludge; GS—granular sludge; M—methanogenic assays; IR—Fe(III)-reducing assays; OL—oleate; Blk—blank assays (no added carbon source); Abiotic Ctr—abiotic controls (without inoculum).

Oleate biodegradation assays were performed in 500 mL serum bottles containing 250 mL bicarbonate-buffered mineral salt medium [25]. The headspace was flushed and pressurized with N_2_/CO_2_ (80:20% *v*/*v*, 1.7 × 10^5^ Pa). Before inoculation, the medium was supplemented with salts and vitamins [25] and reduced with sodium sulfide (0.8 mmol L^−1^). Suspended sludge (SS) from a municipal anaerobic sludge digester (Choupal wastewater treatment plant, Coimbra, Portugal) and granular sludge (GS) from a brewery wastewater treatment plant (Sociedade Central de Cervejas e Bebidas, S.A., Portugal) were used as inocula, at a volatile solids (VS) final concentration of 3 g L^−1^. The specific methanogenic activity (SMA) of the inocula was determined according to Alves et al. [26], and was expressed in volume of methane produced at standard temperature and pressure (STP) conditions per mass unit of VS of inoculum and time (mL g^−1^ day^−1^). For the suspended sludge, SMA values of 12 ± 1 and 463 ± 30 mL g^−1^ day^−1^ were obtained with acetate (30 mmol L^−1^) and H_2_/CO_2_ (80:20% *v*/*v*), respectively. For the granular sludge, acetoclastic and hydrogenotrophic SMA were 199 ± 18 and 611 ± 16 mL g^−1^ day^−1^, respectively. Sodium oleate (≥99%, Fluka) was added as the carbon source at a concentration of 3 mmol L^−1^. A scheme of the experimental procedure applied is shown in Figure 1. Iron-reducing (IR) assays were amended with neutralized Fe(OH)_3_, prepared in the laboratory as described by Lovley and Phillips [27]. The amount of Fe(OH)_3_ added to the assays (100 mmol L^−1^) was approximately 1/3 of the stoichiometric amount needed for complete oleate oxidation. Methanogenic (M) assays were established in the absence of any added external electron acceptor. Blank assays (without added substrate) and abiotic controls (without inoculum) were also prepared (Figure 1). All the assays were made in triplicate and incubations were performed at 37 °C, in the dark, and without agitation. Due to the low solubility of oleate, the collection of representative samples for quantification is difficult and thus, we used the measurement of the products formed over time to follow oleate biodegradation more accurately. Methane was measured in the bottles’ headspace during the experiment, and expressed relatively to the volume of liquid. Volatile fatty acids (VFA) and Fe(II) concentrations were measured over the time. LCFA were quantified at the beginning and at the end of the incubations.

### 2.2. Oleate-Degrading Fe(III)-Reducing Enrichment Cultures

Cultures amended with Fe(OH)_3_ in the previous experiment (IR-SS-OL and IR-GS-OL, Figure 1) were used as inocula for starting oleate-degrading Fe(III)-reducing enrichments. These enrichments were designated SS(x) and GS(x), where x represents the number of transfers. Basal medium was prepared as described above. Fe(OH)_3_ and sodium oleate were added at 75 and 1 mmol L^−1^, respectively, to attain a close-to-stoichiometric Fe(III)/oleate ratio. The cultures were monitored by phase contrast microscopy and Fe(II) concentrations were measured periodically. Successive transfers (10% *v*/*v*) to new medium were performed after observing microbial growth and Fe(III) reduction. Incubations were made at 37 °C, in the dark, and without agitation.

The series developed from suspended sludge (SS, Figure 1) lost its viability after three successive transfers, and thus, this enrichment culture was not further characterized. Physiological characterization of the enrichment culture obtained from granular sludge was performed after 5 successive transfers (GS(5), Figure 1), when a stable enrichment was obtained. Assays were done in triplicate by measuring methane, VFA, and Fe(II) concentrations over time, and LCFA were measured at the beginning and at the end of the experiment. The microbial composition of the enrichment cultures GS(3) and GS(5) was analyzed by DNA extraction and 16S rRNA gene sequencing (Illumina MiSeq Inc., San Diego, CA, USA).

### 2.3. Testing Oleate Degradation by Geobacter Species

Bacteria *Geobacter anodireducens* SD-1^T^ (KCTC 4672T) and *G. bemidjiensis* DSM 16622^T^ were purchased from the Korean Collection for Type Cultures (KCTC, Jeollabuk-do, Korea) and from the Deutsche Sammlung von Mikroorganismen und Zellkulturen (DSMZ, Braunschweig, Germany), respectively. Cultures were grown as described above in duplicate assays, in the presence of ferric citrate and sodium oleate at final concentrations of 55 and 1 mmol L^−1^, respectively. Ferric citrate was chosen as the electron acceptor for these incubations based on the *Geobacter anodireducens* SD-1^T^ characterization made by Sun et al. [28], and also on the DSMZ medium recommended for *G. bemidjiensis* DSM 16622^T^. Ferric citrate was added to the medium from a sterile anaerobic stock solution. A control was prepared with ferric citrate and acetate (10 mmol L^−1^) as the carbon source, and a negative control, containing ferric citrate and oleate but without *Geobacter* cells, was prepared as well. Incubations were performed at 37 °C, statically, and in the dark. Fe(III) reduction was checked by visual inspection.

### 2.4. Analytical Methods

Methane concentration was measured by gas chromatography (Chrompack CP 9000), using a Porapak Q column and a flame ionization detector. N_2_ was used as carrier gas at 30 mL min^−1^. Injection port, column, and detector temperatures were 100, 35, and 220 °C, respectively. VFA were quantified by HPLC (Jasco, Tokyo, Japan) in centrifuged and filtered (0.22 µm) liquid samples. A Chrompack organic analysis column (30 × 6.5 mm) was used at 60 °C, with H_2_SO_4_ (5 mmol L^−1^) as the mobile phase at 0.6 mL min^−1^. The detection was made spectrophotometrically at 210 nm. LCFA were extracted and quantified as previously described by Neves et al. [29]. Briefly, esterification of free fatty acids was performed with propanol in acid medium for 3.5 h at 100 °C. Propyl esters were further extracted with dichloromethane and analyzed in a gas chromatograph (Varian 3800, Agilent, Santa Clara, CA, USA) equipped with a flame ionization detector and a eq. CP-Sil 52 CB 30 m × 0.32 mm × 0.25 µm capillary column (TR-WAX, Teknokroma, Barcelona, Spain). Helium was used as carrier gas at a flow rate of 1.0 mL min^−1^. Initial oven temperature was set at 50 °C for 2 min and a final temperature of 225 °C was attained with a ramp rate of 10 °C min^−1^. Injector and detector temperatures were 220 and 250 °C, respectively. Fe(II) concentration was analyzed in 0.1 mL samples after extraction with HCl (0.5 mol L^−1^) for 1 h [27]. The concentration of HCl-extractable Fe(II) was quantified using a ferrozine solution (1 g L^−1^ in 50 mmol L^−1^ HEPES (4-(2-hydroxyethyl)-1-piperazineethanesulfonic acid, pH 7.5)), by measuring the absorbance of the Fe(II)-ferrozine complex at 562 nm in a Biotech Synergy HT spectrophotometer (BioTek Instruments, Inc., Winooski, VT, USA). After overnight extraction in the dark, total iron was converted to Fe(II) by incubation with hydroxylamine-HCl (1.4 mol L^−1^) for 1 h, and quantified with ferrozine, as described. Calibration standards for both ferrous and total iron were prepared using ferrous ethylenediammonium sulfate and ferric chloride solution, respectively.

### 2.5. Microbial Composition Analysis of Oleate-Degrading Fe(III)-Reducing Enrichment Cultures

Aliquots of well-homogenized sludge were collected and immediately frozen at −20 °C. Total genomic DNA was extracted using the FastDNA SPIN Kit for Soil (MP Biomedicals, Biomedicals, Solon, OH, USA). DNA amplification, Illumina library preparation, amplicon sequencing (Illumina MiSeq, Inc., San Diego, CA, USA), and bioinformatics analysis of the data were performed at the Research and Testing Laboratory (Lubbock, TX, USA). Samples were amplified for sequencing using the universal primer pair 515f and 806r [30], targeting the prokaryotic 16S rRNA gene. Details on the sequencing and bioinformatics data analysis can be found elsewhere [31]. Nucleotide sequences were submitted to the European Nucleotide Archive (ENA) under the accession numbers ERS3774021 to ERS3774023, associated with the study number PRJEB33626. A comparison between operational taxonomic units (OTU) 16S rRNA gene sequences and the NCBI RefSeq_RNA database was performed using the BLASTN alignment tool (http://ncbi.nlm.nih.gov/blast).

### 2.6. Identification of Fatty Acid Degrading Proteins in Geobacter anodireducens and Syntrophomonas zehnderi

Protein FASTA sequences of the complete proteomes of *Syntrophomonas zehnderi* strain OL-4^T^ and *Geobacter anodireducens* SD-1^T^ [32] were obtained from the Uniprot database (taxon identifier: 690567, 2631 proteins) and the NCBI database (GenBank: CP014963.1, CP014964.1), respectively. Functional annotation was performed by submitting FASTA files to the reCOGnizer tool (version 1.2.3, available through GitHub at github.com/iquasere/reCOGnizer and through Bioconda at anaconda.org/bioconda/recognizer) to identify protein-conserved domains related to fatty acid degradation. The search was directed to find the following Clusters of Orthologous Groups (COG): COG1022 (FadD, Long-chain acyl-CoA synthetases (AMP-forming), EC 6.2.1.3), COG0318 (FadD, Acyl-CoA synthetases (AMP-forming)/AMP-acid ligases II)), COG2025 (FadE, Electron transfer flavoprotein, alpha subunit, EC 1.3.8.1), COG1960 (FadE, Acyl-CoA dehydrogenases), COG1024 (FadB, Enoyl-CoA hydratase), COG1250 (FadB, 3-hydroxyacyl-CoA dehydrogenase), and COG0183 (FadA, Acetyl-CoA acetyltransferase).

## 3. Results

### 3.1. Oleate Degradation with Sub-Stoichiometric Fe(III) Concentration by Anaerobic Sludge

At the beginning of the incubations, Fe(II) concentrations around 4 mmol L^−1^ were measured in all the bottles, and total iron concentrations of 105 ± 5 and 115 ± 14 mmol L^−1^ were quantified in the assays with suspended and granular sludge, respectively. The difference between those values is the Fe(III) concentration, which was 101 ± 6 and 111 ± 13 mmol L^−1^ in the bottles with suspended and granular sludge, respectively.

**Figure 2:**
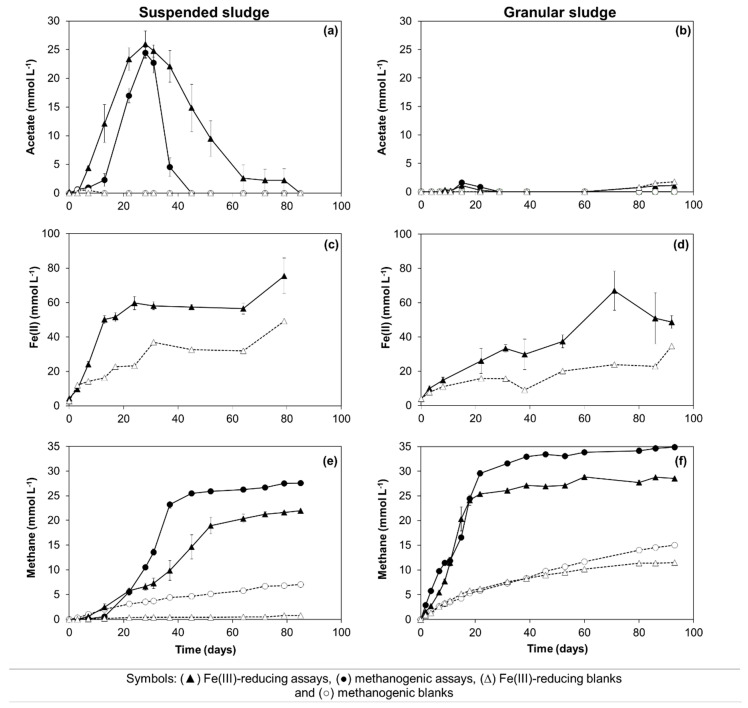
Biodegradation assays performed with sub-stoichiometric Fe(III) concentration: acetate (**a**,**b**), Fe(II) (**c**,**d**), and methane (**e**,**f**) concentrations measured in the assays inoculated with suspended or granular sludge, respectively. Open symbols were used for the blanks.

Oleate biodegradation was verified in all the conditions studied, i.e., initial oleate concentrations decreased to undetectable levels (data not shown) in the assays inoculated with suspended and granular sludge, both in the presence and absence of Fe(III) (Figure 2). In the experiments with suspended sludge, oleate was mainly converted to acetate during the first 28 days of incubation (Figure 2a), reaching a cumulative concentration close to the theoretical stoichiometric values, i.e., 26 ± 2 and 24 ± 1 mmol L^−1^ in the iron-reducing (IR) and methanogenic (M) assays, respectively (Figure 2a, Table 1—Equations 6 and 8). Acetate accumulation started earlier in the IR assays (Figure 2a), and was associated with Fe(III) reduction (Figure 2c). Indeed, Fe(II) concentrations increased up to 60 ± 4 mmol L^−1^ during the first 24 days of incubation. A Fe(II) concentration of 23 mmol L^−1^ was also measured at the same time point in the blank assays amended with Fe(III) (Figure 2c), suggesting the occurrence of Fe(III)-reduction due to residual substrate oxidation. Therefore, assuming an equivalent Fe(III) consumption due to residual substrate in the assays amended with oleate, approximately 37 mmol L^−1^ of Fe(III) was possibly used for oleate oxidation to acetate coupled to Fe(III) reduction, corresponding to an oleate consumption of about 1.2 mmol L^−1^ (Table 1—Equation (6)).

Methane production in the IR assays started in the first 3 days of incubation, while a lag phase of 13 days was observed in the M assays (Figure 2e). After 28 days of incubation, methane concentrations of 6.6 ± 0.7 and 10.5 ± 0.3 mmol L^−1^ were achieved in the IR and M assays, respectively, (Figure 2e). Considering the stoichiometry of oleate conversion to acetate and hydrogen (Table 1—Equation (1)), and further H_2_ consumption by hydrogenotrophic methanogens (Table 1—Equation (5)), a methane production of approximately 11.25 mmol L^−1^ was expected from hydrogen (Table 1—Equation (8)), which is close to the value measured in the M assays. If 1/3 of the generated electrons were used for Fe(III) reduction, only 7.5 mmol L^−1^ of methane could be expected, which is also close to the experimental value obtained in the IR assays. Therefore, these results show that almost all the added oleate was oxidized to acetate in the first 28 days of incubation, and the electrons resulting from this conversion were used for methanogenesis in the M assays, while in the IR assays, the electrons were used both for Fe(III)-reduction and methanogenesis.

Acetate consumption by the suspended sludge occurred only after day 28 and appeared to be closely related to methane production (Figure 2a,e), which was faster in the methanogenic assays (i.e., after day 28, the methane production rate was 1.4 ± 0.1 and 0.6 ± 0.0 mmol L^−1^ d^−1^ in the M and IR assays, respectively). At the end of the IR and M assays, the methane produced accounted for 57% and 72% of the theoretical value expected, respectively (Table 2, Table 1—Equation (9)). Acetate was not detected in the blank assays (Figure 2a), and methane was produced at low amounts in the blanks prepared without Fe(III) addition, possibly resulting from the degradation of residual substrate and endogenous cell decay (Figure 2e).

In the experiments with granular sludge, oleate was mainly converted to methane, and acetate was rarely detected in the medium (Figure 2b,f). Similar methane production rates were observed in the IR and M assays, as shown by the slopes of the cumulative methane production curves (Figure 2f), and the maximum methane production accounted for 75% and 91% of the theoretical expected value, respectively (Table 2). In the IR assays, Fe(III) reduction and methane production occurred simultaneously (Figure 2d,f).

Methane production was faster in the assays with granular sludge (Figure 2f) than in the one with suspended sludge (Figure 2e). Methane production was not detected in the abiotic controls during all the experiments, and neither was Fe(III) reduction (data not shown).

### 3.2. Oleate-Degrading Fe(III)-Reducing Enrichment Cultures

**Figure 3:**
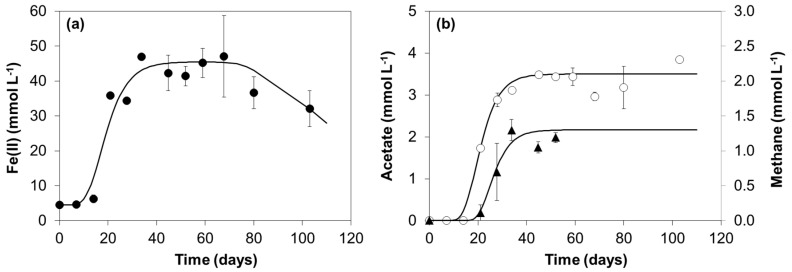
Fe(II) (●) (**a**), and acetate (○) and methane (▲) (**b**) concentrations measured during the incubation of culture GS(5) with oleate and Fe(OH)_3_.

A microbial community capable of oleate degradation and Fe(III) reduction was enriched from granular sludge. In culture GS(5), obtained after five successive transfers, approximately 42 ± 0.4 mmol L^−1^ of Fe(II) accumulated in the medium after 34 days of incubation (Figure 3a), representing approximately 57% of the total Fe(III) added, and oleate was not present in the vials at the end of the incubations. The nonmagnetic reddish-brown Fe(OH)_3_ changed to a black fine-grained precipitate that was attracted to a magnet (Figure S1). Acetate accumulated in the medium up to an average value of 3.4 ± 0.3 mmol L^−1^ (Figure 3b) and cumulative methane production reached a maximum value of 1.2 ± 0.1 mmol L^−1^ (Figure 3b).

Microbial community analysis showed the predominance of bacteria assigned to *Geobacter* and *Syntrophomonas* genera, with relative abundances of 83–89% and 3–6%, respectively (Table 3). The 16S rRNA gene sequences, obtained by Illumina MiSeq (approximately 291 bp length), that were assigned to *Geobacter*, were aligned to those in the NCBI Reference Sequence Database (RNA) and showed 100% identity to *G. anodireducens* strain SD-1^T^, and 99.6% identity to *G. soli* and *G. sulfurreducens* (Table 3 and Table S1). For the *Syntrophomonas* sequences, an identity of 95.3% with *Syntrophomonas zehnderi* OL-4^T^ was obtained (Table 3). The archaeal population was composed by members of the *Methanobacterium* genus, accounting for less than 10% of the total microbial community.

### 3.3. Testing Oleate Biodegradability by Geobacter Species

Pure cultures of *G. anodireducens* SD-1^T^ and *G. bemidjiensis* DSM 16622^T^ were incubated with oleate in the presence of ferric citrate, to investigate the ability of these *Geobacter* strains to degrade oleate. *G. anodireducens* was chosen because it is the closest relative of the *Geobacter* strain present in cultures GS(3) and GS(5), and *G. bemidjiensis* because, based on information from the genome analysis [35], this bacterium possesses genes encoding a long-chain fatty acyl-CoA dehydrogenase (*fadE)*, and can potentially have the ability to perform LCFA degradation.

Initially, all cultures presented a dark brown color, typically from ferric citrate. Over the time, for both *Geobacter* strains, no obvious color change could be visually detected in the bottles containing oleate (bottles I and II, Figure S2a,b), as well as in the negative control (bottle IV, Figure S2a,b), while in the control assays with acetate, the color of the medium changed to light yellow (bottle III, Figure S2a,b). These observations point to the occurrence of Fe(III) reduction with acetate, but not with oleate, suggesting that these two strains are not capable of oxidizing oleate coupled to Fe(III) reduction.

### 3.4. Identification of Fatty Acid Degrading Proteins in Geobacter anodireducens and Syntrophomonas zehnderi

The occurrence of genes coding for enzymes involved in fatty acid degradation in the genomes of *Geobacter anodireducens* and *Syntrophomonas zehnderi* was investigated. This revealed the presence of key enzymes participating in the fatty acid degradation pathway in both microbial genomes, although the number of gene copies associated with this pathway was much higher in *S. zehnderi* (69 gene copies) than in *G. anodireducens* (11 gene copies) (Tables S2 and S3).

*S. zehnderi* possesses thirteen genes coding for acyl-CoA synthetases/ligases (FadD), from which three are long-chain fatty acid-specific; twenty three acyl-CoA dehydrogenases (FadE), from which three are short-chain-specific (EC 1.3.8.1); seventeen enoyl-CoA hydratases and seven 3-hydroxyacyl-CoA dehydrogenase (FadB); and nine acetyl-CoA acetyltransferases (FadA) (Table S3). *G. anodireducens* also contains genes coding for the complete fatty acid degradation pathway, although only two gene copies were found to be long-chain fatty acid-specific, namely two long-chain acyl-CoA synthetases (FadD) (Table S2). Two additional gene copies were assigned to FadD, three to FadE, two to Fad B (1 enoyl-CoA hydratase and one 3-hydroxybutyryl-CoA dehydrogenase), and two to FadA.

## 4. Discussion

The effect of Fe(III) on methanogenic LCFA biodegradation is scarcely studied and several different scenarios can be hypothesized, namely LCFA biodegradation by IRB, or by syntrophic bacteria in close relationship with H_2_/formate- and/or acetate-utilizing IRB as syntrophic partners. Fe(III) concentration appears as a crucial factor, and thus, in this work, sub-stoichiometric and stoichiometric concentrations were applied. Two different microbial communities, exhibiting different methanogenic activity and aggregation structure, were investigated considering that differences in the original microbial composition and aggregation might also have an important role.

The solubility of Fe(III) decreases from acidic to circumneutral pH conditions and thus, in our assays, the redox couple Fe(OH)_3_/Fe^2+^ rather than Fe^3+^/Fe^2+^ is expected to prevail [16,34]. The redox potential of the couple Fe(OH)_3_/Fe^2+^ is more negative than that of Fe^3+^/Fe^2+^, i.e., −0.236 V and +0.772 V, respectively [34]. Hence, Fe(III) reduction is thermodynamically less favorable at neutral pH. Standard Gibbs free energy changes (Δ*G*^0′^) were calculated at 25 °C and pH 7 for acetate, hydrogen, or oleate conversion and, for each substrate, the values obtained under Fe(III)-reducing conditions, considering the redox couple Fe(OH)_3_/Fe^2+^ (Table 1—reactions 2, 4, and 7), were similar to those under methanogenic conditions (Table 1—reactions 3, 5, and 9), showing no thermodynamic advantage of Fe(III) reduction over methanogenesis.

Nevertheless, the presence of Fe(III) triggered a faster oleate biodegradation by the suspended sludge, as shown by the faster acetate accumulation verified in the IR relative to the M assays, especially during the first 13 days (Figure 2a). Considering that Fe(II) and methane were produced concomitantly with close-to-stoichiometric acetate accumulation in the IR assays (Figure 2c,e), oleate oxidation was most likely performed by syntrophic bacteria in close relationship with both hydrogen-consuming IRB and methanogens. LCFA conversion by syntrophic bacteria and sulfate- or sulfonate-reducing hydrogen-consuming bacteria was previously reported by Sousa et al. [36] and Salvador et al. [31], respectively, but syntrophic interaction between LCFA-degrading bacteria and IRB was not described before. Dissimilatory Fe(III) reduction proceeded at a faster rate than hydrogenotrophic methanogenesis (Figure 2c,e), which possibly represented an advantage for the syntrophic relationships, thus accelerating oleate oxidation.

In our incubations, IRB did not out-compete hydrogenotrophic methanogens, but instead, these two trophic groups were capable of coexisting using the same substrate (hydrogen/formate). Moreover, the presence of the IRB appears to have accelerated hydrogenotrophic methanogenesis, since methane production started within 3 days in the IR assays and a 13 day lag phase was observed in the M assays (Figure 2e). Coexistence of methanogens and sulfate-reducing bacteria was reported before [37,38,39], highlighting a metabolic flexibility which may have also influenced the results from this study.

Despite the positive effect of IRB activity on oleate oxidation and on hydrogenotrophic methanogenesis, the complete conversion of oleate to methane was limited in this community by the rate of acetate conversion, both in the presence and absence of Fe(III) (Figure 2a). In the M assays, acetate accumulated up to stoichiometric values during the first 28 days, but thereafter, its degradation was fast, which suggests the occurrence of an inhibitory effect of oleate towards the acetoclastic methanogens. This suggestion is also supported by the results of the activity test, where a similar acetate concentration was completely converted to methane in approximately 4 days (101 h, Figure S3). In fact, acetoclastic methanogens have been reported as the most sensitive microorganisms to LCFA toxicity in anaerobic processes [9,40,41]. In the IR assays, acetate was mostly utilized by the methanogens, possibly because the ferric hydroxide concentration needed to oxidize the accumulated acetate was 208 mmol L^−1^ (Table 1—reaction 2) and only 40 mmol L^−1^ was available in the medium at day 28. However, acetate consumption was slower than in the M assays (Figure 2e), suggesting the inhibition of acetoclastic methanogens by iron.

In the incubations with granular sludge, methanogens and Fe(III)-reducing microorganisms also coexisted (Figure 2d,f), but in this case, the effect of Fe(III) on oleate degradation was not observed, since acetate did not accumulate in the medium and methane was produced at similar rates in the IR and M assays, without the occurrence of lag phases preceding the onset of methane production in both conditions. This effect may be related to the higher activity of the methanogenic community in this sludge, which could effectively compete for the available substrates, or the granular structure of the sludge might have provided some protection against toxicity to the methanogenic archaea [42]. Moreover, the structure of the granules also facilitates syntrophic relations by promoting physical proximity between syntrophic partners [43]. Nevertheless, it is important to consider that granules tend to disintegrate in anaerobic digesters treating high LCFA loads [41] and thus, these protective effects may be lost.

Based on our results, the presence of Fe(III) or exogenous Fe(III) supplementation to anaerobic digesters treating LCFA-rich waste/wastewater has a high potential for practical applications, contributing to faster LCFA degradation, particularly when the methanogenic activity is low. In this way, effective treatment of LCFA-rich wastewater will likely be accomplished, avoiding process inhibition and failure. From an applied point of view, the possible occurrence of abiotic reactions during AD should be considered, since these will play an important role in iron speciation and bioavailability. For example, in the presence of sulfide, Fe(II) may precipitate as ferrous sulfide (FeS), while Fe(III) may chemically oxidize sulfide to elemental sulfur, with itself being reduced to Fe(II) and ensuing precipitation as FeS [16]. Iron complexation and precipitation with organic and inorganic ligands is also frequent [16].

The presence of *Syntrophomonas*, a genus of syntrophic fatty acids degraders, in the enriched cultures developed from granular sludge (Table 3) suggests that these bacteria were degrading oleate. Still, the most abundant microorganism in the microbial community was assigned to the *Geobacter* genus (83–89% relative abundance, Table 3), which is known to encompass species able to grow with acetate or hydrogen while reducing Fe(III) [44]. Despite the fact that genome analysis of *Geobacter bemidjiensis* and other subsurface *Geobacter* species showed the existence of genes encoding a long-chain fatty acyl-CoA dehydrogenase, FadE, suggesting the potential for LCFA metabolism [35], this potential is not widespread amongst the *Geobacter* genus, since FadE-coding genes are absent from the genome of *G. sulfurreducens* and *G. metallireducens* [35]. On the other hand, the presence of genes coding for enzymes involved in LCFA degradation does not imply that the microorganism is able to degrade these compounds. For example, *G. bemidjiensis* was not able to utilize caproate [45], and Coates et al. [15] tested six different *Geobacter* strains with palmitate, but none were capable of degrading this saturated LCFA. In this work, we evaluated oleate degradation by pure cultures of *G. anodireducens* and *G. bemidjiensis,* but the two strains did not grow with oleate coupled to Fe(III) reduction (Figure S2). Nevertheless, the *G. anodireducens* genome contains genes coding for enzymes potentially involved in the degradation of fatty acids, including two long-chain acyl-CoA synthetases/ligases, which may indicate the potential of this microorganism to activate long-chain fatty acids (Table S2). However, the only 3-hydroxyacyl-CoA dehydrogenase found in the *G. anodireducens* genome was annotated as 3-hydroxybutyryl-CoA dehydrogenase and is probably short-chain-specific. In comparison, a higher number of genes related to fatty acid metabolism could be found in the genome of *Syntrophomonas zehnderi* (Table S3), the LCFA-degrading specialist that was present in the enrichment culture together with *G. anodireducens*, which may reflect the high evolutionary adaptation of this bacterium to perform LCFA degradation [46].

Considering that *Methanobacterium* species were the only methanogens in culture GS(5), and that members of this genus reduce carbon dioxide with hydrogen, the methane produced by culture GS(5) (Figure 3b) accounted for 32% of the electrons that could derive from oleate oxidation to acetate (Table 1—Equation (1)), and for 9% of all the electrons that could be produced if acetate oxidation to CO_2_ was considered as well (Equation (10) [33]):

C_2_H_3_O_2_^−^ + 4 H_2_O → 2 HCO_3_^−^ + 9 H^+^ + 8 e^−^ (Equation 10)

Since the oleate added was almost completely consumed, the hydrogen that was not directed to methane was probably coupled to Fe(III) reduction by *Geobacter*. Taking all the information together, it seems likely that *S. zehnderi* and not *G. anodireducens* degrades oleate in this culture, and that both *Methanobacterium* and *Geobacter* species work as hydrogen scavengers in syntrophic oleate degradation catalyzed by *Syntrophomonas*. Nevertheless, methane production lags behind in comparison with Fe(II) and acetate production (Figure 3), suggesting a competitive advantage of *Geobacter*. This bacterium was probably using acetate as well, since maximum concentrations around 3.4 mmol L^−1^ were detected in the medium, while 9 mmol L^−1^ was expected from the oxidation of approximately 1 mmol L^−1^ oleate (Table 1—Equation (1)), which indicates that acetate was partially consumed. However, maximum Fe(II) concentrations (42 ± 0.4 mmol L^−1^) were lower than expected, i.e., 20.4 mmol L^−1^ from the H_2_ that was not used for methane plus 45.0 mmol L^−1^ from the oxidation of 5.6 mmol L^−1^ of acetate. This was probably related with the formation of a black fine-grained precipitate with magnetic properties, resembling magnetite, observed in the enriched culture GS(5) (Figure S1). Magnetite is an iron(II,III) oxide that can be formed from secondary reactions between ferric hydroxide and the Fe(II) resulting from ferric hydroxide reduction by IRB [47]. The Fe(II) extraction method used in this work (extraction with 0.5 mol L^−1^ HCl) is not able to recover more than 0.5% of the Fe(II) from magnetite [48], thus hindering the quantification of Fe(II) that was formed. Additionally, the formation of insoluble and less accessible complexes containing the Fe(III) such as magnetite, make Fe(III) reduction more difficult for the bacteria. This may be the reason why acetate was not completely oxidized and still remained in the medium at the end of the incubation (Figure 3b).

## 5. Conclusions

The presence of sub-stoichiometric Fe(III) amounts accelerated oleate biodegradation by the suspended sludge, but had no noticeable effect in the assays with granular sludge, highlighting the importance of the inoculum sludge. Enrichment cultures growing on oleate and iron selected a microbial consortium mainly formed by *Syntrophomonas, Geobacter*, and *Methanobacterium*, showing novel microbial interactions in LCFA oxidation.

## Figures and Tables

**Table 1:** Possible reactions involved in oleate biodegradation under methanogenic or Fe(III)-reducing conditions.

Reaction No.	Condition ^1^	Reactant	Equation	Δ*G*^0′^ (kJ Reaction^−1^) ^2^
1	-	Oleate	β-oxidation of oleate	
			C_18_H_33_O_2_^−^ + 16 H_2_O → 9 C_2_H_3_O_2_^−^ + 15 H_2_ + 8 H^+^	+325.7
2	IR	Acetate	Acetate oxidation coupled to Fe(III) reduction	
			C_2_H_3_O_2_^−^ + 8 Fe(OH)_3_ + 15 H^+^ → 2 HCO_3_^−^ + 8 Fe^2+^ + 20 H_2_O	−32.9
3	M	Acetate	Methanogenesis from acetate	
			C_2_H_3_O_2_^−^ + H_2_O → HCO_3_^−^ + CH_4_	−31.0
4	IR	Hydrogen	Hydrogen oxidation coupled to Fe(III) reduction	
			H_2_ + 2 Fe(OH)_3_ + 4 H^+^ → 2 Fe^2+^ + 6 H_2_O	−34.4
5	M	Hydrogen	Methanogenesis from hydrogen	
			H_2_ + 0.25 HCO_3_^−^ + 0.25 H^+^ → 0.25 CH_4_ + 0.75 H_2_O	−33.9
6 = 1 + 4	IR	Oleate	Oleate oxidation to acetate coupled to Fe(III) reduction	
			C_18_H_33_O_2_^−^ + 30 Fe(OH)_3_ + 52 H^+^ → 9 C_2_H_3_O_2_^−^ + 30 Fe^2+^ + 74 H_2_O	−189.9
7 = 1 + 2 + 4	IR	Oleate	Overall reaction for oleate oxidation to CO_2_ coupled to Fe(III) reduction	
			C_18_H_33_O_2_^−^ + 102 Fe(OH)_3_ + 187 H^+^ → 18 HCO_3_^−^ + 102 Fe^2+^ + 254 H_2_O	−485.9
8 = 1 + 5	M	Oleate	Oleate oxidation to acetate coupled to hydrogenotrophic methanogenesis	
			C_18_H_33_O_2_^−^ + 3.75 HCO_3_^−^ + 4.75 H_2_O → 9 C_2_H_3_O_2_^−^ + 3.75 CH_4_ + 4.25 H^+^	−182.8
9 = 1 + 3 + 5	M	Oleate	Overall reaction for oleate oxidation to methane	
			C_18_H_33_O_2_^−^ + 13.75 H_2_O → 5.25 HCO_3_^−^ + 12.75 CH_4_ + 4.25 H^+^	−461.8

^1^ IR—iron-reducing; M—methanogenic. ^2^ Gibbs free energy changes were calculated at standard conditions (solute concentrations of 1 mol L^−1^, gas partial pressure of 10^5^ Pa, T = 25 °C, pH 7), based on the values presented by Thauer et al. [33], Widdel et al. [34], and Salvador et al. [31].

**Table 2:** Maximum cumulative methane production and methane recovery in the Fe(III)-reducing and methanogenic assays.

Assay ^1^	Max. Cumulative Methane Production (mmol L^−1^)	Methane Recovery (%) ^2^
IR-SS-OL	22 ± 0	57 ± 1
M-SS-OL	28 ± 0	72 ± 0
IR-GS-OL	29 ± 0	75 ± 1
M-GS-OL	35 ± 0	91 ± 1

^1^ IR—iron-reducing; M—methanogenic; SS—suspended sludge; GR—granular sludge; OL—oleate. ^2^ Methane recovery (%) = (Methane produced/theoretical methane concentration expected) × 100. The theoretical methane concentration expected was 38.25 mmol L^−1^, corresponding to the complete conversion of 3 mmol L^−1^ oleate to methane.

**Table 3:** Phylogenetic affiliation of the 16S rRNA gene sequences identified at the genus level in the libraries from enrichment cultures GS(3) and GS(5).

Taxonomic Identification ^1^	Relative Abundance (%)			Closest Cultured Relatives Based on 16S rRNA Gene Identity ^3^	16S rRNA Gene Identity (%) ^3^	Accession No.
	GS(3)	GS(5)-a ^2^	GS(5)-b ^2^			
*Geobacter*	86.4	89.4	83.2	*Geobacter anodireducens* SD-1	100.0	NR_126282.1
*Syntrophomonas*	5.9	3.4	4.3	*Syntrophomonas zehnderi* OL-4	95.3	NR_044008.1
Unclassified (*Clostridiales*)	2.7	0.0	0.0	*Colidextribacter massiliensis* Marseille-P3083	93.7	NR_147375.1
*Aminiphilus*	1.0	0.4	1.2	*Aminiphilus circumscriptus* ILE-2	99.6	NR_043061.1
*Methanobacterium*	0.2	5.0	9.7	*Methanobacterium formicicum* MF ^5^ *Methanobacterium beijingense* 8-2 ^6^	100.0 100.0	NR_115168.1 NR_028202.1
Other ^4^	3.9	1.8	1.6	-	-	-

^1^ Taxonomic identification at the genus level based on 16S rRNA gene sequences of approximately 291 bp length obtained by Illumina MiSeq. ^2^ Results of duplicate samples. ^3^ Results of sequence alignment by using BLASTN towards the RefSeq_rna database. ^4^ OTU with relative abundance < 1% were included in Other. ^5^ Closest relative for the OTU in sample GS(3). ^6^ Closest relative for the OTU in samples GS(5)-a and GS(5)-b.

## Supplementary Materials

The following are available online at https://www.mdpi.com/2076-2607/8/9/1375/s1, Figure S1: Pictures of the Fe(III)-reducing enrichment cultures GS(5) after Fe(OH)_3_ reduction, showing the accumulation of a black precipitate that was attracted to a magnet (probably magnetite), Figure S2: Pictures of *Geobacter anodireducens* SD-1^T^ (a) and *Geobacter bemidjiensis* DSM 16622^T^ (b) batch incubations with ferric citrate and oleate (bottles I and II) or acetate (bottle III). Bottle IV is the negative control, prepared with oleate and Fe(III) (without inoculum), Figure S3: Cumulative methane production from acetate measured in the SMA tests performed with the suspended sludge, Table S1: Results of sequence alignment of the operational taxonomic units (OTU) assigned to *Geobacter* genus towards NCBI RefSeq_RNA database, by using BLAST, Table S2: Proteins associated with fatty-acid degradation encoded in the genome of *Geobacter anodireducens* SD-1^T^. Protein annotation was obtained from the NCBI database. COG functional categories and EC numbers were obtained by running reGOGnizer, Table S3: Proteins associated with fatty-acid degradation encoded in the genome of *Syntrophomonas zehnderi* strain OL-4^T^. Protein names and sequences were obtained from the Uniprot database, COG functional categories and EC numbers were obtained by running reGOGnizer.
