# Supplementary material for: Effect of Sub-Stoichiometric Fe(III) Amounts on LCFA Degradation by Methanogenic Communities
Source: Microorganisms. 2020 Sep 7;8(9):1375. doi: 10.3390/microorganisms8091375 (PMC7564256; doi:10.3390/microorganisms8091375)
Supplement: Supplementary file 1 [file microorganisms-08-01375-s001.zip › microorganisms-902092 suppl final/Supplementary Information_final/Cavaleiro et al_Supplementary Information_final.docx]

**Effect of Sub-Stoichiometric Fe(III) Amounts on LCFA Degradation by Methanogenic Communities**

**Ana J. Cavaleiro ^1,^*, Ana P. Guedes ^1,¥^, Sérgio A. Silva ^1^, Ana L. Arantes ^1^, João C. Sequeira ^1^, Andreia F. Salvador ^1^, Diana Z. Sousa ^1, 2^, Alfons J. M. Stams ^1, 2^ and M. Madalena Alves ^1^**

^1^ Centre of Biological Engineering, University of Minho, Campus de Gualtar, 4710-057 Braga, Portugal; sergiosilva@ceb.uminho.pt (S.A.S.), analuisa.pereira@ceb.uminho.pt (A.L.A.); jsequeira@ceb.uminho.pt (J.C.S.); asalvador@ceb.uminho.pt (A.F.S.); diana.sousa@wur.nl (D.Z.S.); fons.stams@wur.nl (A.J.M.S.); madalena.alves@deb.uminho.pt (M.M.A.)

^2^ Laboratory of Microbiology, Wageningen University & Research, Wageningen, 6708 WE, The Netherlands

^¥^ Present address: Agricultural Superior School of Ponte de Lima, Polytechnic Institute of Viana do Castelo, Refóios do Lima, 4990-706 Ponte de Lima, Portugal; guedesana@esa.ipvc.pt

***** Correspondence: acavaleiro@deb.uminho.pt; Tel.: +35 1253604423

Received: 31 July 2020; Accepted: 4 September 2020; Published: date

**SUPPLEMENTARY INFORMATION**


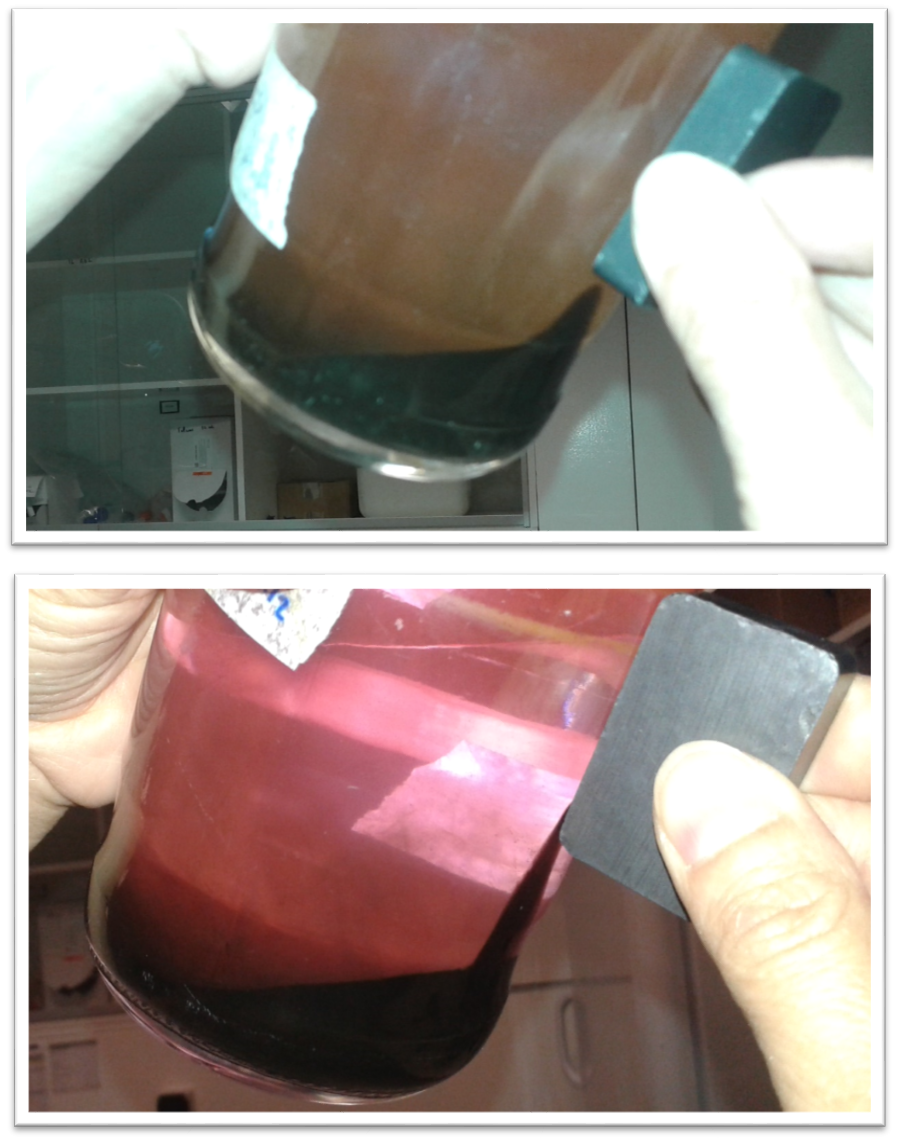


**Figure S1.** Pictures of the Fe(III)-reducing enrichment cultures GS(5) after Fe(OH)_3_ reduction, showing the accumulation of a black precipitate that was attracted to a magnet (probably magnetite).


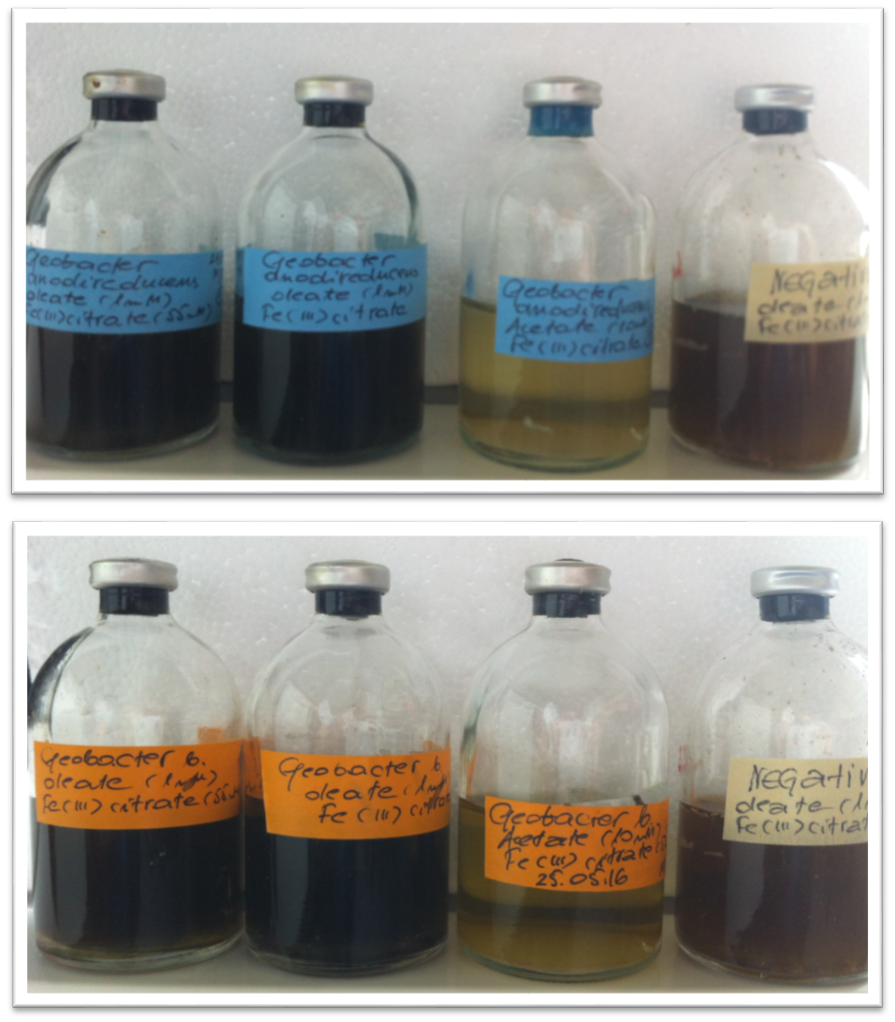


**(a)**

**(b)**

**(I)**

**(II)**

**(III)**

**(IV)**

**(I)**

**(II)**

**(III)**

**(IV)**

**Figure S2.** Pictures of *Geobacter anodireducens* SD-1^T^ (a) and *Geobacter bemidjiensis* DSM 16622^T^ (b) batch incubations with ferric citrate and oleate (bottles I and II) or acetate (bottle III). Bottle IV is the negative control, prepared with oleate and Fe(III) (without inoculum).


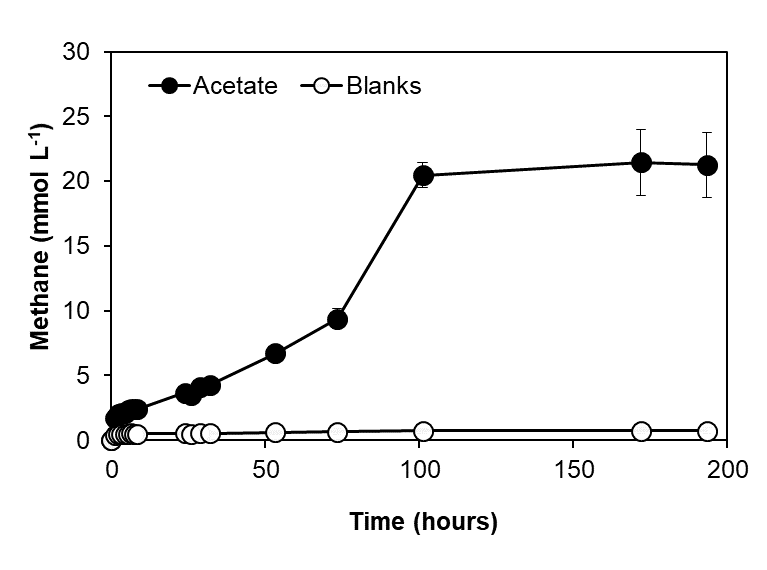


**Figure S3.** Cumulative methane production from acetate measured in the SMA tests performed with the suspended sludge.

**Table S1.** Results of sequence alignment of the operational taxonomic units (OTU) assigned to *Geobacter* genus towards NCBI RefSeq_RNA database, by using BLAST.

| **Description** | **Identity (%)** | **Accession number** |
| --- | --- | --- |
| *Geobacter anodireducens* SD-1^T^ | 100.00 | NR_126282.1 |
| *Geobacter soli* GSS01^T^ | 99.60 | NR_134039.1 |
| *Geobacter sulfurreducens* PCA^T^ | 99.60 | NR_075009.1 |
| *Geobacter sulfurreducens* | 99.60 | NR_029179.1 |
| *Geobacter sulfurreducens* subsp. *ethanolicus* OSK2A^T^ | 99.21 | NR_132673.1 |
| *Geobacter metallireducens* GS-15^T^ | 96.84 | NR_075011.1 |
| *Geobacter pickeringii* G13^T^ | 96.84 | NR_025895.1 |
| *Geobacter metallireducens* GS-15^T^ | 96.84 | NR_025895.1 |
| *Geobacter grbiciae* TACP-2^T^ | 96.84 | NR_104561.1 |
| *Geobacter luticola* OSK6^T^ | 96.44 | NR_114303.1 |
| *Geobacter hydrogenophilus* H2^T^ | 96.46 | NR_025974.1 |
| *Geobacter daltonii* FRC-32^T^ | 96.05 | NR_074916.1 |
| *Geobacter daltonii* FRC-32^T^ | 96.05 | NR_116402.1 |
| *Geobacter toluenoxydans* TMJ1^T^ | 96.05 | NR_116428.1 |

**Table S2.** Proteins associated with fatty-acid degradation encoded in the genome of *Geobacter anodireducens* SD-1^T^. Protein annotation was obtained from the NCBI database (Sun et al. 2016). COG functional categories and EC numbers were obtained by running reGOGnizer **[EXCEL FILE]**.

**Table S3.** Proteins associated with fatty-acid degradation encoded in the genome of *Syntrophomonas zehnderi* strain OL-4^T^. Protein names and sequences were obtained from the Uniprot database, COG functional categories and EC numbers were obtained by running reGOGnizer **[EXCEL FILE]**.
